# Design and experiment of spoon shaped clamping chrysanthemum seedling transplanting mechanism

**DOI:** 10.3389/fpls.2026.1777945

**Published:** 2026-03-02

**Authors:** Qian Wu, Rongyan Wang, Yaoqi Feng, Zijian Ding, Junjie Qian, Gang Zhao

**Affiliations:** School of Engineering, Anhui Agricultural University, Hefei, China

**Keywords:** chrysanthemum seedlings, kinematic analysis, parameter optimization, trajectory simulation, transplanting mechanism

## Abstract

This work aims at solving the problems of susceptibility of chrysanthemum seedlings with multiple fibrous roots to damage, poor adaptability of traditional clamping type transplanting machines, and unstable transplanting uprightness. According to the agronomic requirements of chrysanthemum transplanting and morphological characteristics of its seedlings, a spoon shaped clamping transplanting device of chrysanthemum seedlings was designed and optimized. A kinematic model was established based on its working principle, and a human-computer interaction analysis interface of the transplanting device was developed based on MATLAB, and the influence of key structural parameters on transplanting performance was analyzed through this interface, and the optimal parameter combination of the mechanism was determined. The transplanting trajectory was verified by simulation and field experiment. The results showed that the actual trajectory and simulation trajectory of spoon shaped clamping transplanting device were in good agreement with the theoretical design trajectory. Under the operating conditions of crank speed 67 r·min^-1^ and forward speed 0.19 m·s^-1^, the qualified rate of transplanting angle was 84.20%, the qualified rate of transplanting depth was 90.0%, the coefficient of variation (CV) of planting spacing was 5.8%. The transplanting spacing is stable, the erect degree is high, and the transplanting meets the agronomic requirements. This study can provide reference for the development of mechanized transplanting equipment for multi-fibrous root seedlings.

## 1 Introduction

Chrysanthemum is a officinal and food crop, rich in a variety of essential trace elements, with eyesight, anti-inflammatory and other effects, as well as having both high medicinal and economic values (Roosta et al., 2017). With the improvement of health awareness, chrysanthemum demand and planting scale continue to expand, so that China’s chrysanthemum planting area has reached approximately 53,000 hectares. However, the industry and the market of chrysanthemum transplanting machinery is still under a blank and seriously restricted the development. At present, manual transplanting has problems such as high labor intensity, low efficiency, high cost and inconsistent transplanting depth, which increases the difficulty of later management and harvesting (Yu et al., 2014). The existing transplanting machines are mainly divided into chain clamp type, dibble type, gripper type and duckbill type, according to the characteristics of seedling throwing and seedling sending of planting mechanism (Li et al., 2022). For example, semi-automatic pot (dry soil) seedling transplanting machine 2ZL of FLW manufacturer adopts chain clamp transmission, manually places seedlings into chain clamps, moves to designated positions along with chain, and then transplants them into soil. The machine has simple structure and low manufacturing cost, but the transplanting efficiency needs to be improved and the phenomenon of missing seedlings is obvious (Liu et al., 2025). The dibble type creates a hole in the soil into which a seedling is dropped. This mechanism requires precise synchronization between hole creation and seedling release, sometimes leading to poor uprightness (Na et al., 2021). The gripper type mechanisms, employing finger like clamps to grasp the seedling plug or stem, they risk damaging tender stems, especially for fibrous stemmed plants like chrysanthemums (Han et al., 2019; Prakash and Ilangkumaran, 2024). The duckbill planter is widely used at present. Its transmission mode mainly includes multi-link type and planetary gear train type. The multi-link type structure is simple and various operation trajectories can be realized through parameter adjustment. The manufacturing cost is low but the vibration of the machine body is caused by high inertia force during operation (Yin et al., 2020). The planetary gear rotary device designed by Yu et al. (2015) overcomes the problem of high vibration but, due to the influence of planting track, the qualified rate of verticality is not good. Zhang (2025) developed the software for the multi-pose motion trajectory of rice pot seedling transplanting device by MATLAB, based on the analysis of key pose points and genetic algorithm, and computed of the key parameters. Through ADAMS kinematic simulation, Li et al. (2025) obtained the velocity curves of seedling picking rod and seedling receiving rod, and determined the optimal position of seedling throwing and planting. Zhang (2021) and Jin et al. (2012) analyzed the influence of different mechanisms on the trajectory through ADAMS simulation and developed a planting device on film that conforms to the predetermined trajectory. The semi-automatic or automatic transplanting system integrating machine vision and robotic arms has also been applied for precise picking and placement of seedlings, but its high cost and complexity limit its application in small and medium-sized operations (Ankit and Sanjay, 2024; Han et al., 2025). Various transplanting machines adapt to seedlings with different characteristics. At present, there are a few researches on chrysanthemum mechanical transplanting.

This study focuses on tea chrysanthemum (hereinafter referred to as chrysanthemum) seedlings. Seedlings are derived from the rhizomes or stems of the mother plant, and usually have 3~5 functional leaves and multiple root systems. In order to ensure the survival rate of seedlings, it is necessary to maintain the integrity of roots and leaves during transplanting. Because of lack of root protection during the process of planting by means of clamping type transplanting machine the roots are easy to be damaged, due to friction between roots and the soil or planting mechanism, which affects subsequent growth of transplanted seedlings. In contrast, spoon type mechanisms utilize concave surfaces to cradle and support the root from below, minimizing root disturbance. Existing spoon designs, however, primarily focus on conveying the root ball and often lack active stem stabilization, which can result in seedling tilting or stem-leaf interference during the extraction and planting phases for tall, upright seedlings (Abhijit et al., 2025). Therefore, it is necessary to develop a transplanting machine suitable for chrysanthemum seedling which can not only protect the roots of seedlings but also ensure the transplanting depth, verticality and spacing.

In order to meet the requirements of transplanting trajectory and erect posture of chrysanthemum seedlings, this study combined chrysanthemum transplanting agronomic and biological characteristics of seedlings, so that a spoon shaped clamping chrysanthemum seedling transplanting device was designed. Based on MATLAB, the software interface of human-computer interactive visual assistant analysis of planting mechanism was developed to analyze the influence of each structural parameter on transplanting trajectory. Transplanting trajectory, seedling position, transplanting angle and depth, the optimal structure and movement parameters were optimized, and the rationality of the mechanism was verified by field experiments. This can provide reference for theoretical research and innovative design of chrysanthemum transplantation machines.

## 2 Materials

### 2.1 Agricultural requirements for transplanting chrysanthemum seedlings

**Figure 1:**
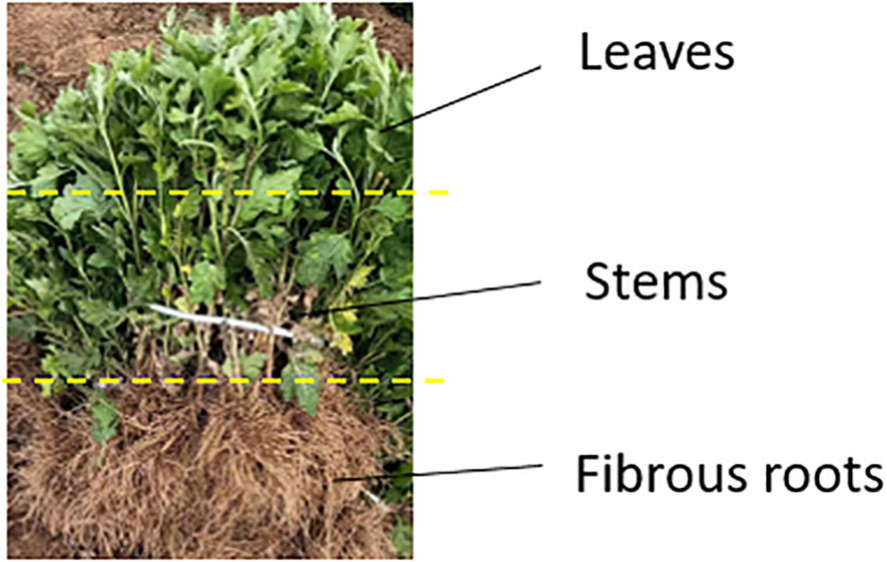
Chrysanthemum seedlings.

Chrysanthemum seedlings are composed of three parts: fibrous roots, stems, and leaves, with a total length of 200~300 mm. The diameter of the main stem is about 5 mm, and the root system is well-developed and dispersed, with a fibrous root length of 60~100 mm. The chrysanthemum seedlings are shown in **Figure 1**.

**Figure 2:**
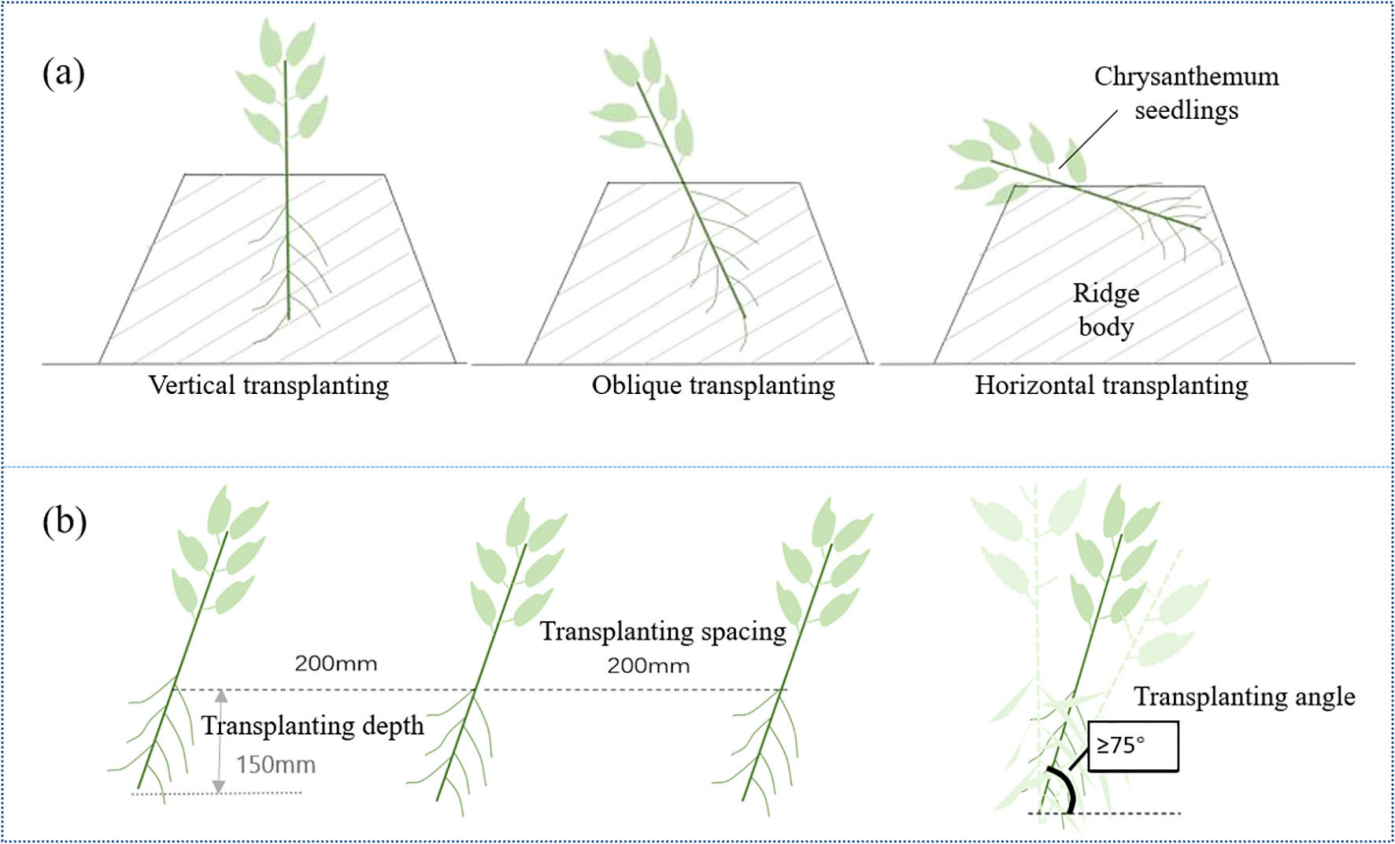
Agronomic model of chrysanthemum seedling transplantation. **(a)** Transplanting mode **(b)** Transplanting details.

The yield of chrysanthemum was mainly affected by transplanting depth, density and method. Ridge cultivation was adopted for chrysanthemum seedlings. Taking *Gongju* as an example, ridge width was 350 mm, and transplanting spacing was 200 mm, according to soil fertility conditions. There are three common transplanting methods: vertical transplanting; oblique transplanting; horizontal transplanting (**Figure 2a**). Vertical transplanting is beneficial to reduce seedling stage, does not need to adapt to root posture for a long time, and can adapt to soil faster. In actual planting, the angle between stem and horizontal plane is within 80° ± 5°, which can be regarded as vertical transplanting. When transplanting, the roots should be completely buried in the soil, and the depth should be appropriate. Too shallow roots do not contribute to water conservation and affects survival; too deep roots will lead to low temperature and hinder seedling growth (Li et al., 2023; Mu et al., 2023; Hou et al., 2019). According to the biological characteristics of chrysanthemum seedlings and the requirements of transplanting method, the vertical transplanting method was adopted. The parameters are set as transplanting depth of 150 mm, transplanting angle ≥75° and transplanting spacing of 200 mm (**Figure 2b**).

### 2.2 Transplanting machine structure and main technical parameters

**Figure 3:**
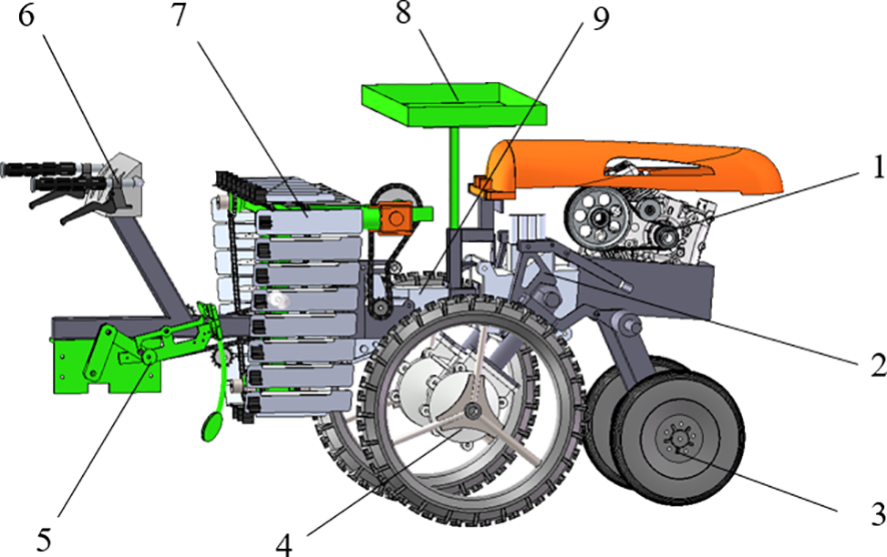
Structure and composition of spoon shaped clamping chrysanthemum seedling transplanting machine [1) Engine 2) Height adjustment device 3) Front support wheels 4) Rear driving wheels 5) Planting device 6) Control system 7) Seedling guide plate 8) Seedling tray 9) Gearbox].

Spoon shaped clamping chrysanthemum seedling transplanting machine (**Figure 3**) is mainly composed of an engine, a height adjusting device, two front support wheels, two rear driving wheels, a planting device, a control system, a seedling guide plate, a seedling tray and a gearbox.

The chrysanthemum transplanting machine adopts belt transmission to transmit engine power to gearbox, and then drives seedling guide plate and planting device through wheel chain mechanism. During operation, chrysanthemum seedlings are manually placed in rubber clips on the upper part of the seedling guide plate, and the roots are kept outward and exposed for 30~40 mm. The seedling guide plate transports the seedlings via chain drive to the planting device, which then grips the seedling roots for insertion into the soil. The planting device adopts crank-rocker parallel transmission mechanism, and its movement track is composed of rotation around crank shaft and opening and closing movement of clamping device. Diversified track can be realized by adjusting crank speed and rod length. The spoon shaped clamping device cooperates with cam mechanism to precisely control the clamping opening and closing angle and seedling posture. Based on the stem diameter, root morphology and other characteristics of chrysanthemum seedlings, the opening size of planting device was established in order that the clamping device could adapt to the seedling morphology and ensure their integrity during transplanting. By precisely coordinating the action sequence of seedling feeding mechanism, crank-rocker and spoon shaped clamping device, the continuous operational flow of seedling picking, clamping and planting was established. The main technical parameters of the spoon shaped clamping chrysanthemum seedling transplanting machine are shown in **Table 1**.

**Table 1:** Main parameters of spoon shaped clamping chrysanthemum seedling transplanting machine.

Parameters	Value
Engine power (kW)	4~7
Forward speed (km·h^−1^)	0.3~1.5
Number of work lines	1
Applicable ridge height (mm)	250~350
Machine wheelbase (mm)	700
Transplanting spacing (mm)	160~250
Transplanting depth (mm)	100~200

### 2.3 Structure and working principle of transplanting device

**Figure 4:**
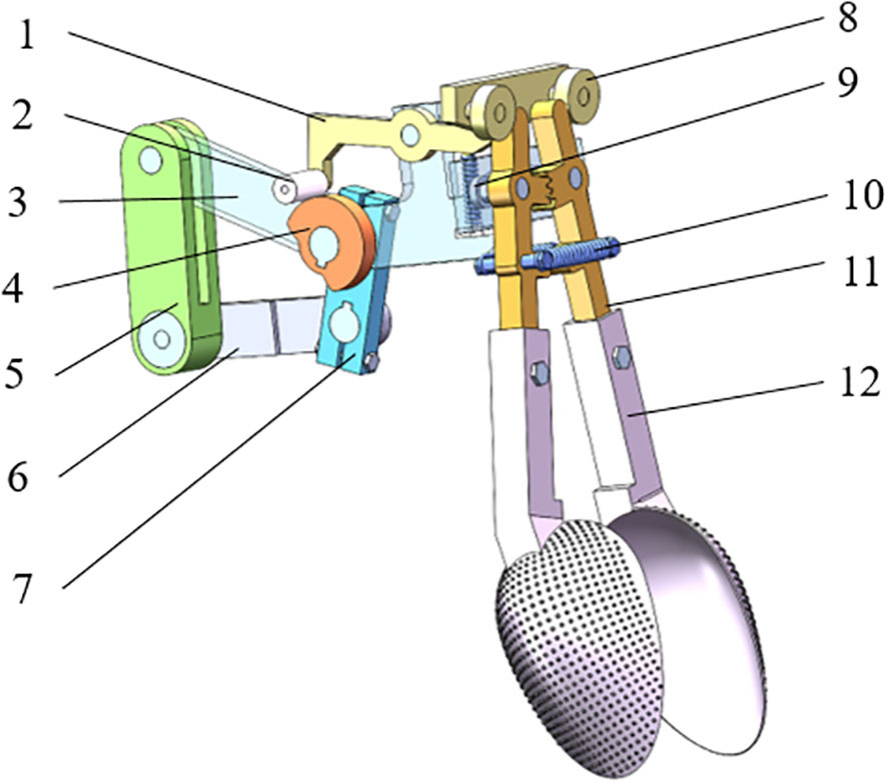
Three dimensional structure of transplanting device [1) Swing push rod 2) Cam roller 3) Connection rod 4) Cam 5) Rocker 6) Frame 7) Crank 8) Roller 9) Swing push rod spring 10) Opening and closing spring 11) Opening and closing part 12) Spoon shaped clamping device].

Chrysanthemum seedling transplanting is composed of seedling picking, clamping and planting. The transplanting device is the core component to realize insertion of seedlings, and its performance directly affects transplanting quality (Sharma and Khar, 2024; Zhang et al., 2022). The transplanting device has a three-dimensional structure, as shown in **Figure 4**, and mainly comprises a crank-rocker, a connection rod, a frame, a cam, a swing push rod, a cam roller, an opening and closing part, a spoon shaped clamping device, a swing push rod spring and an opening and closing spring. The swing push rod is hinged with the connecting frame rod through a shaft and can rotate around the central hole. The front end and the rear end of the swing push rod are respectively equipped with rollers in contact with the opening and closing parts. The spoon shaped clamping device consists of a spoon-shaped curved surface and a rod piece, and realizes opening and closing under the combined action of a swing push rod, a cam and an opening and closing part. The outer part of the spoon shaped curved surface is in direct contact with the soil, and the inner concave space is used for accommodating and enveloping the roots of chrysanthemum seedlings.

**Figure 5:**
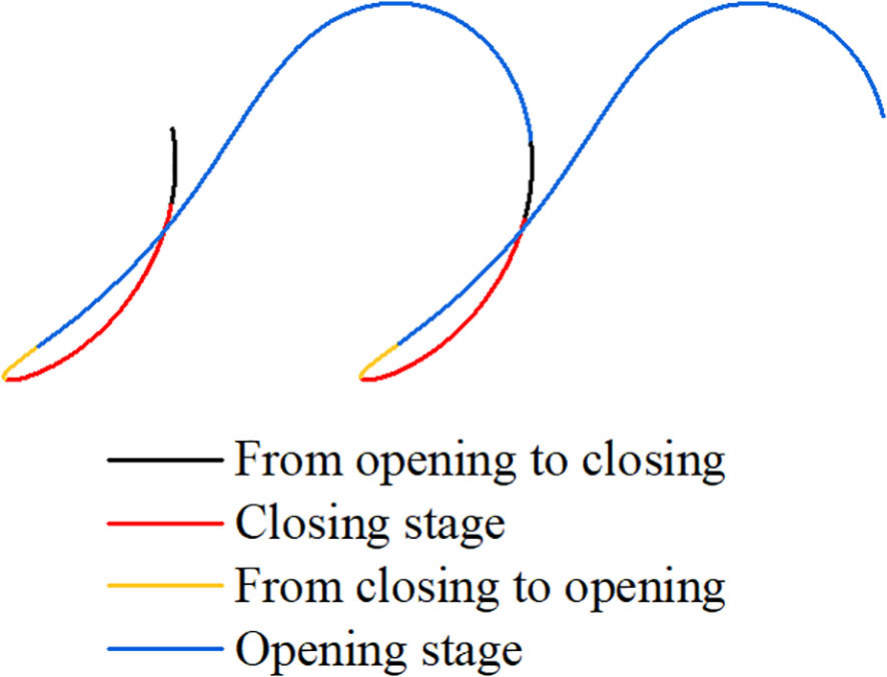
Stages corresponding to the seedling clamping point during transplanting.

The working performance of transplanting device depends directly on its transplanting trajectory characteristics (Yang et al., 2022; Jiang et al., 2017). During transplanting, the crank rotates clockwise to drive the connection rod to rotate around the crank shaft, which drives the cam to rotate around the same axis and rotate with reference to the cam roller. Cam profile can be divided into a high radius section and a low radius section according to its distance difference from center. When the cam roller rotates from the low radius section to come into contact with the high radius section, the distance between the rear end of the swing push rod and the cam center increases, forcing the swing push rod to rotate clockwise around the cam center, and the spring at the lower part is compressed. At the same time, the roller at the front end of the swing push rod extrudes the opening and closing part inwards to rotate around the cam center, so as to drive the spoon shaped clamping device to open and complete the seedling throwing action; at this time, the spring on the opening and closing part is stretched. As the cam continues to rotate, when the cam roller comes into contacts with the low radius section again, the distance between the rear end of the swing push rod and the cam center decreases, and the spring at the lower end is released. Under the action of the compression force of the spring on the opening and closing part, this rotates reversely to push the roller to return to its original position, and under the joint action of the three groups of springs, the swing push rod is driven to rotate counterclockwise around the cam center. Thus, the spoon shaped clamping device is closed and seedling clamping is realized. The cam rotates continuously to drive the swing push rod to rotate clockwise and counterclockwise alternately, so that the spoon shaped clamping device periodically executes opening and closing actions to accomplish a continuous transplanting operation. **Figure 5** shows the schematic diagram of each stage corresponding to the seedling clamping point during the transplanting operation, wherein, the crank rotates for one cycle *t* = 1.05 s, and the rotation speed of crank is calculated to be 66.7 r·min^-1^. When the average transplanting spacing is 200 mm, the machine forward speed is calculated to be 0.19 m·s^-1^.

**Figure 6:**
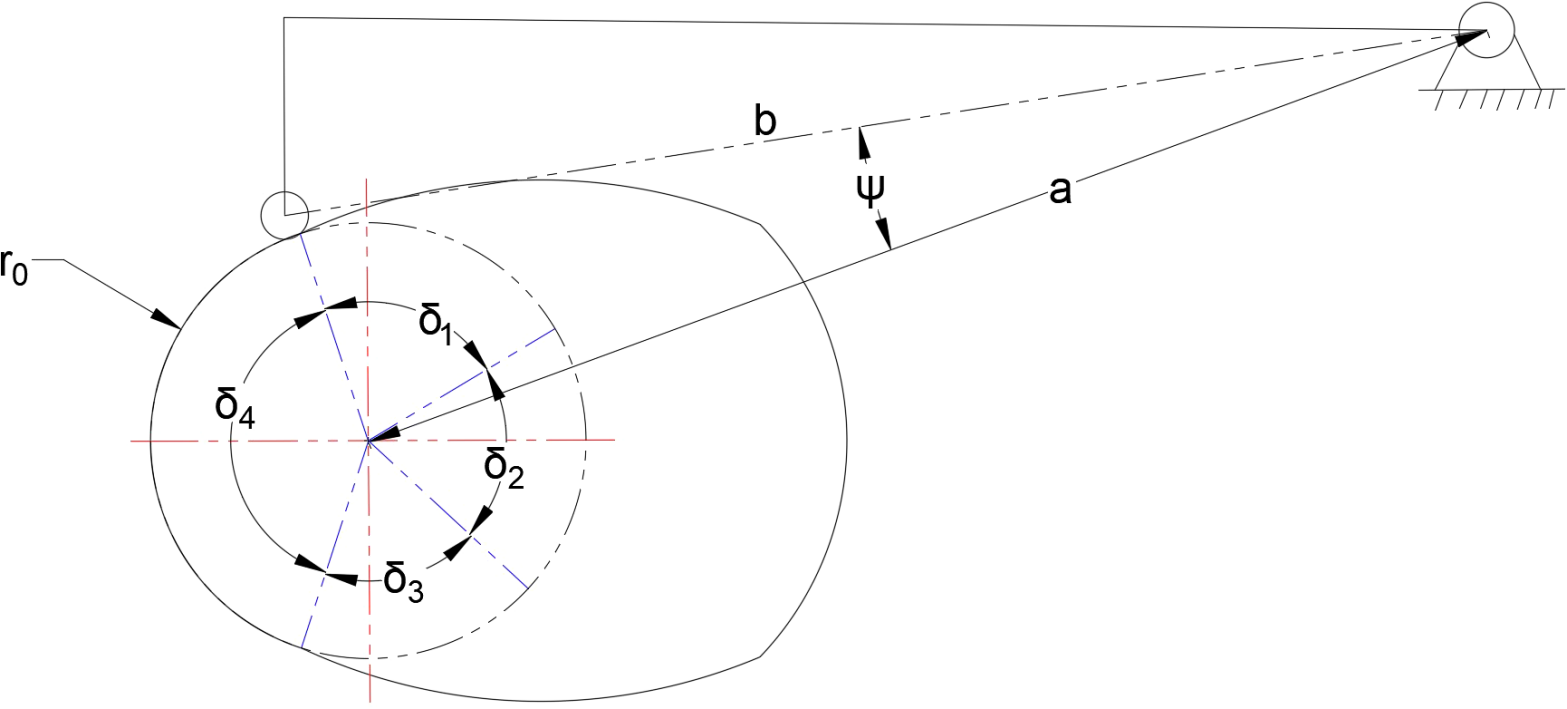
Schematic diagram of disc cam structure.

The transplanting device comprises a standard roller, a swing disc cam, and its structure is shown in **Figure 6**. According to the requirements of transplanting spacing and depth, the coordinates of crank at seedling clamping point and seedling releasing point are determined by visual program, and parameters such as cam far rest angle, near rest angle, pushing movement angle and return movement angle are calculated in combination with transmission efficiency and opening distance of bottom end of clamping device. The specific values are shown in **Table 2**.

**Table 2:** Cam design parameters.

Parameter	Value
Base circle radius/r_0_ (mm)	25.0
Push movement angle/δ_1_ (°)	25
Far rest angle/δ_2_ (°)	82
Return movement angle/δ_3_ (°)	25
Near rest angle δ_4_ (°)	227
Stroke (maximum swing angle of swing push rod)/ψ(°)	8
Distance from center/a (mm)	79.53
Swing rod length/b (mm)	70.51

**Figure 7:**
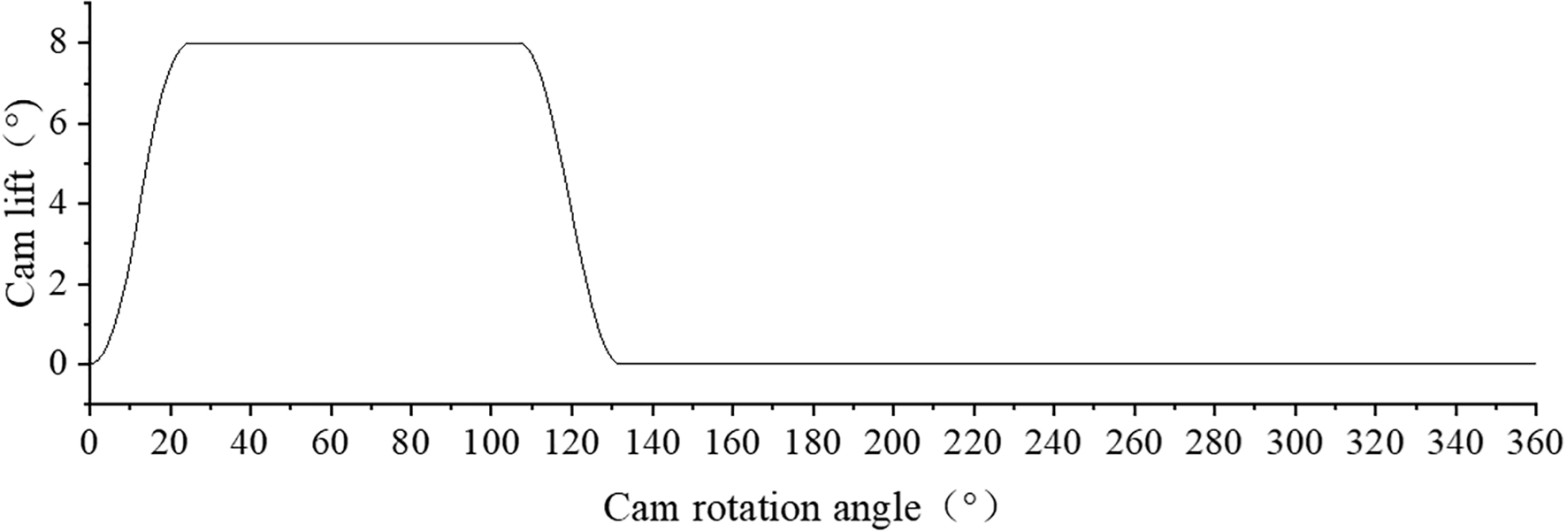
Cam profile curve.

In order to ensure the smoothness of the swing push rod motion, sinusoidal acceleration motion law is adopted for both push stroke and return stroke. The cam profile is shown in **Figure 7**.

### 2.4 Kinematical modeling

**Figure 8:**
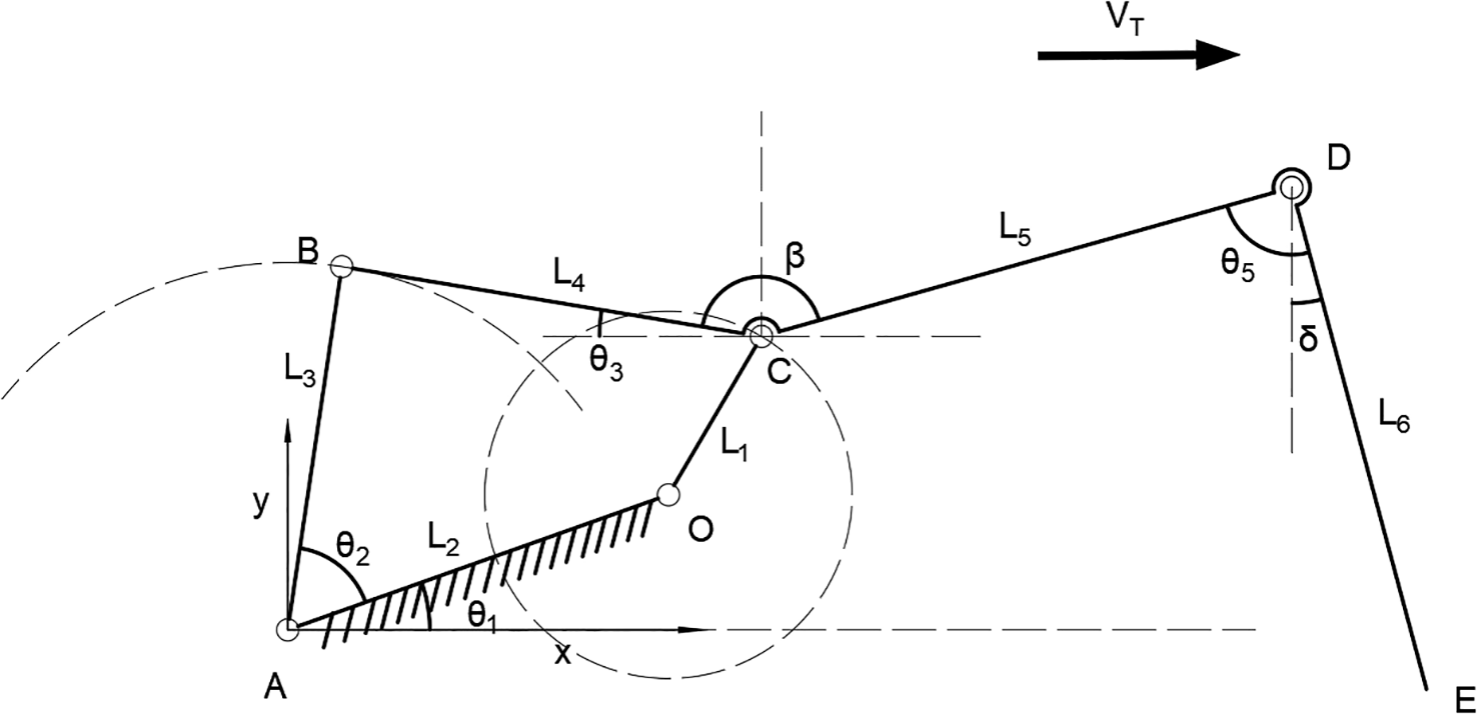
Kinematic model of transplanting device.

The kinematic model of the transplanting device, is shown in **Figure 8**, where A is the origin of coordinate system, X axis is the horizontal direction (X axis positive direction and mechanism forward speed direction are the same), Y axis is the vertical direction. In the **Figure 8**, crank OC is the driving part, AO is the frame, AB is the rocker, BC is the connection rod, BCD is the hole, CD is the extension rod, DE is the seedling clamping device, the angle between OA and the horizontal direction is θ_1_, the angle between OA and AB is θ_2_, the angle between BC and the horizontal direction is θ_3_, the angle between OA and the horizontal direction is θ_4_, the angle between CD and DE is θ_5_, the angle between BC and CD is *β*, the angle between DE and the vertical direction is *δ*, the length of crank OC is L_1_, the length of frame AO is L_2_, the length of rocker AB is L_3_, the length of connection rod BC is L_4_, the length of connection mechanism CD is L_5_, and the length of clamping mechanism DE is L_6_. The whole mechanism moves horizontally towards right at V_T_.

During the transplanting operation, the crank OC of the driving part rotates clockwise, and the displacement equation for point O(*X*_O_,*Y*_O_) is Equation 1:

(1) $$\{\begin{array}{c} X_{\text{O}}=L_{4}\cdot cos\theta_{1}+\nu t\\ Y_{\text{O}}=L_{4}\cdot sin\theta_{1} \end{array}$$

The displacement equation for point C(*X*_C_,*Y*_C_) is Equation 2:

(2) $$\{\begin{array}{c} X_{\text{C}}=x_{\text{B}}+L_{2}\cdot cos\;\theta_{3}\\ Y_{\text{C}}=y_{\text{B}}+L_{2}\cdot sin\;\theta_{3} \end{array}$$

According to the crank rocker mechanism, the vector equation is established as Equation 3:

(3) $$\{\begin{array}{c} \vec{AO}+\vec{OC}=\vec{AB}+\vec{BC}\\ \vec{OE}=\vec{OC}+\vec{CD}+\vec{DE} \end{array}$$

The displacement Equation 4 for point B(*X*_B_,*Y*_B_) is obtained:

(4) $$\{\begin{array}{l} X_{\text{B}}=L_{3}\cdot cos\;\left(\theta_{1}+\theta_{2}\right)+\nu t\\ Y_{\text{B}}=L_{3}\cdot sin\;\left(\theta_{1}+\theta_{2}\right) \end{array}$$

The displacement equation for point D(*X*_D_,*Y*_D_) is obtained as Equation 5:

(5) $$\left\{ \begin{array}{l} X_{D}=X_{C}+L_{5}\cdot \cos(\pi -\theta_{3}-\beta )\\ =L_{3}\cdot \cos(\theta_{1}+\theta_{2})+L_{2}\cdot \cos\theta_{3}+L_{5}\cdot \cos(\pi -\theta_{3}-\beta )\\ Y_{D}=Y_{C}+L_{5}\cdot \sin(\pi -\theta_{3}-\beta )\\ =L_{3}\cdot \sin(\theta_{1}+\theta_{2})+L_{2}\cdot \sin\theta_{3}+L_{5}\cdot \sin(\pi -\theta_{3}-\beta ) \end{array}\right.$$

The displacement equation for point E(*X*_E_,*Y*_E_) is Equation 6:

(6) $$\{\begin{array}{l} X_{\text{E}}=X_{\text{D}}+L_{6}\cdot sin\delta \\ =X_{\text{D}}+L_{6}\cdot sin\left(\theta_{5}+\pi /2-\theta_{3}-\beta \right)\\ X_{\text{E}}=Y_{\text{D}}-L_{6}\cdot cos\delta \\ =Y_{\text{D}}-L_{6}\cdot cos\left(\theta_{5}+\pi /2-\theta_{3}-\beta \right) \end{array}$$

## 3 Methods

### 3.1 Theoretical analysis of transplanting trajectory

**Figure 9:**
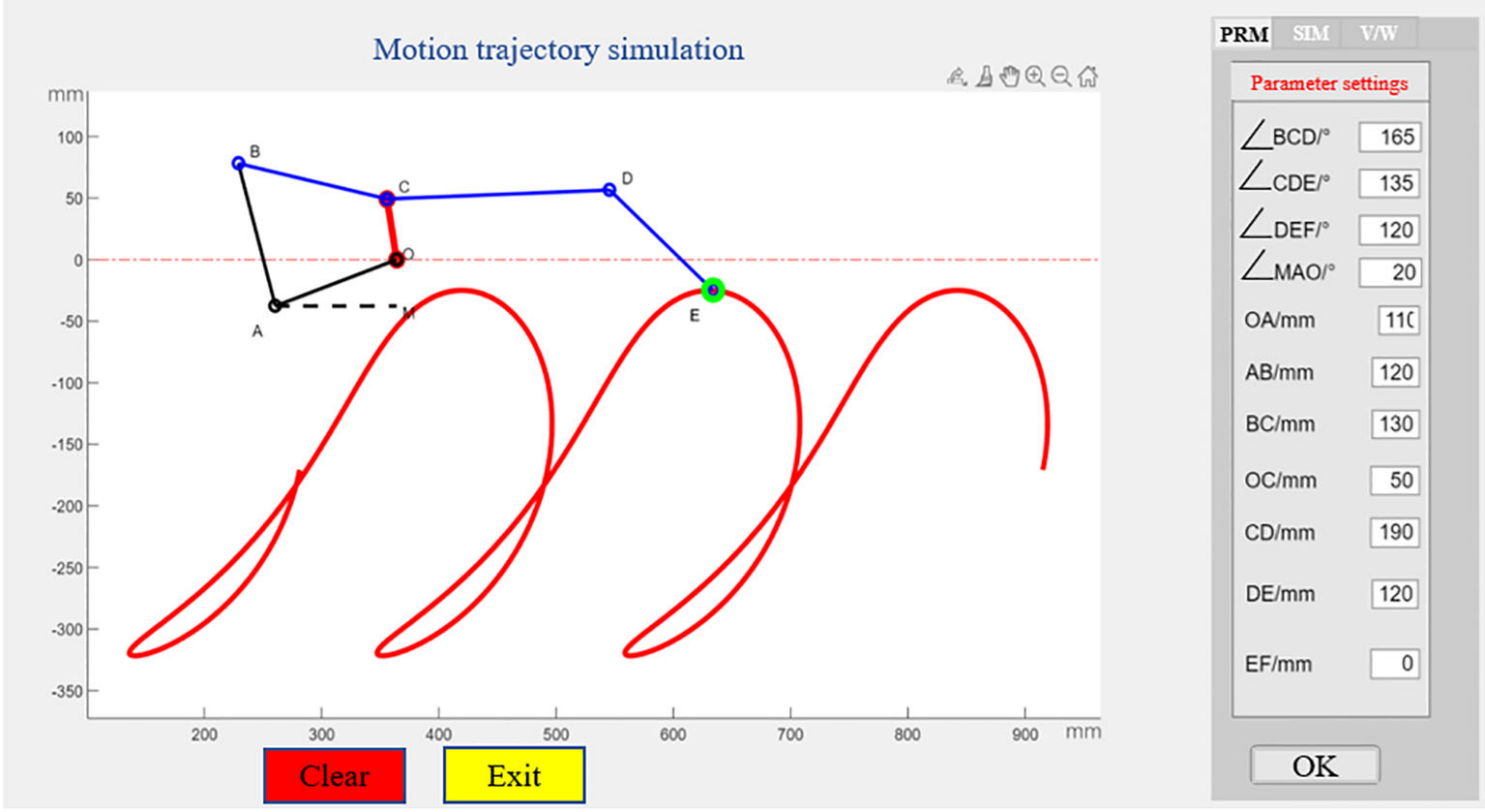
Human-computer interaction visual interface of transplanting device.

Based on the established kinematics model, a human-computer interactive visual assistant interface of transplanting device was developed based on MATLAB GUI module, which was used to analyze the motion state and trajectory of transplanting device, in order to optimize and solve its structural and motion parameters. The graphical user interface (GUI) contains a mechanism motion image display area, parameter input area, control panel area, crank rotation speed and forward speed analysis area, and parameter output area (Xin et al., 2024; Liao et al., 2015; Cai et al., 2019). The known parameters are frame height, transplanting depth and transplanting spacing, and the determined parameters include crank rotation speed, forward speed of transplanting device and conditions of crank-rocker mechanism. The display area can show the mechanism configuration and the movement track of the seedling clamping point E under specific parameters. A coordinate system is established with O point as origin, Y axis and X axis as vertical direction and horizontal direction respectively. The coordinates of hinged points of the mechanism in the coordinate system can be updated and displayed in real time, so as to facilitate the measurement of key parameters such as transplanting depth, transplanting angle and displacement. By adjusting the crank rotation speed, the user can observe the state of the mechanism in different phases and obtain the coordinates of each hinged point in real time. Through the coordinates of them pionts and geometric relationship, the angle and position relationship between each rod of the mechanism can be calculated, and then it is judged whether the movement track of the mechanism meets the agronomic requirements, as shown in **Figure 9**.

Kinematic simulation analysis of mechanism parameters is carried out based on MATLAB platform, and the trajectory of seedling clamping point E is obtained, and then the influence law of each parameter on trajectory shape is explored. On this basis, the parameters are optimized to obtain the optimal parameter combination and its corresponding trajectory, which provides a basis for the subsequent structure size optimization. Combined with the transplanting device size, movement characteristics and agronomic requirements of transplanting, the initial value range of parameters was determined. The transplanting device should meet the following constraints: (1) The horizontal distance between the seedling clamping point E and the origin of coordinate system A is about 400 mm, and the vertical distance is about 100 mm, so as to ensure that the seedling can be erect when moving to the seedling picking position and realize reliable clamping; (2) When the seedling clamping point E reaches the lowest point of the track, it is about 100 mm away from the ridge surface to ensure that the transplanting depth meets the requirements; (3) When the seedling clamping point E is at the lowest position, the angle between the seedling clamping mechanism DE and the Y axis should be kept within 80° ± 5° to meet the requirement of verticality; (4) Sufficient space should be reserved between transplanting device and seedling clamping point E to enable movement for crank-rocker mechanism and avoid interference with seedling feeding mechanism. After preliminary screening, the parameter values shown in **Table 3** were obtained.

**Table 3:** Parameter values of transplanting device.

Parameter	Value		
Crank OC length/L_1_	50 mm	60 mm	70 mm
Frame AO length/L_2_	100 mm	110 mm	120 mm
BC length of connection rod/L_3_	120 mm	130 mm	140 mm
Rocker AB length/L_4_	90 mm	100 mm	110 mm
CD length of extension rod/L_5_	140 mm	150 mm	160 mm
Angle between AO and X-axis/θ_1_	15°	20°	25°
Angle between BC and CD/β	150°	155°	160°
Angle between CD and DE/θ_5_	85°	90°	95°

**Figure 10:**
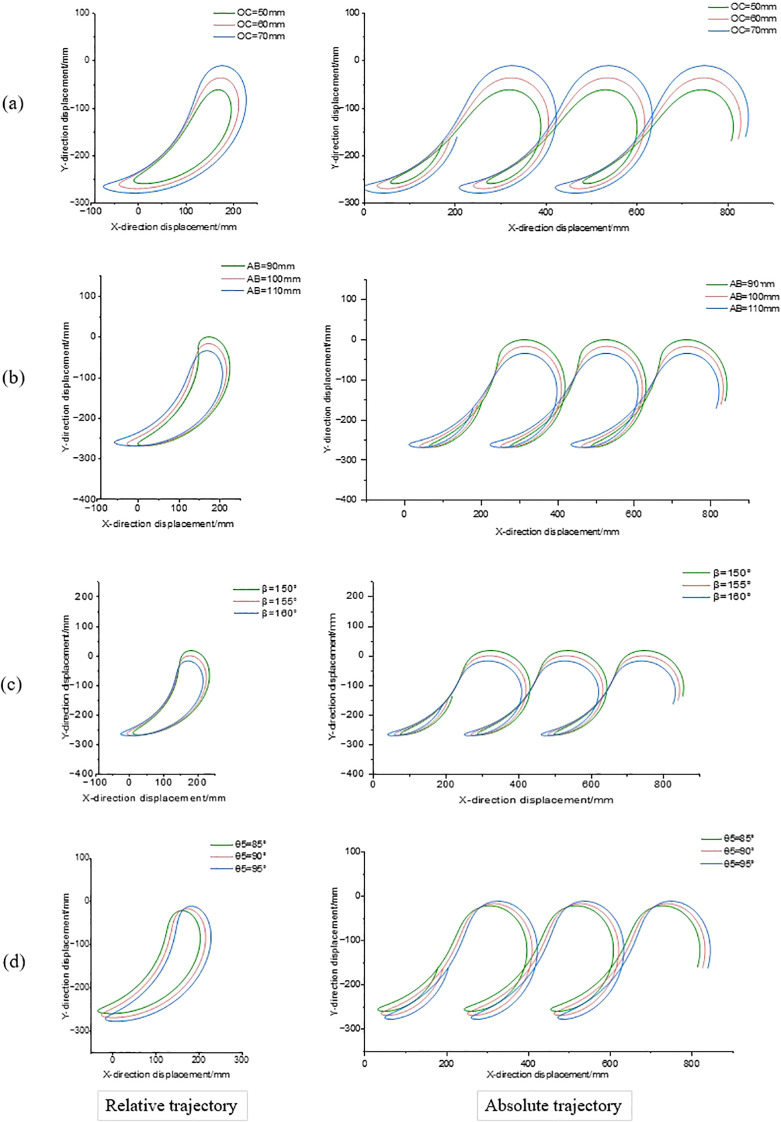
Influence of transplanting device parameters on the trajectory of seedling clamping points. **(a)** The motion trajectory of oc as a variable **(b)** The motion trajectory of AB as a variable **(c)** The motion trajectory of β as a variable **(d)** The motion trajectory of θ5 as a variable.

Through theoretical calculations and simulation analysis, different values of the 8 parameters shown in **Table 3** may affect the transplanting performance. The effects of 8 parameters of transplanting device on its relative and absolute motion trajectories were analyzed by single factor experiment. By observing the track and inclination angle of seedling clamping, the influence of each parameter on transplanting depth and angle was explored. The relative trajectory corresponds to the running trajectory when the crank rotation speed is 66.7 r·min^-1^ and the machine forward speed is 0; the absolute trajectory corresponds to the running trajectory when the crank rotation speed is 66.7 r·min^-1^ and the machine forward speed is 0.19 m·s^-1^. In the simulation experiment, only one parameter was changed at a time, and while the other parameters were kept at intermediate values. **Figure 10** shows the results of four mechanism parameters that have significant effects on transplanting performance.

According to the relative trajectory shown in **Figures 10a**, it can be seen that with the increase of crank OC length L_1_, the vertical distance between the lowest point of the relative movement trajectory of the transplanting device and the ridge surface gradually increases, indicating that the transplanting depth increases accordingly. From the absolute movement trajectory, it can be observed that the angle between trajectory and horizontal direction decreases gradually with the increase of L_1_, so that, the transplanting inclination angle decreases. In order to ensure the transplanting verticality, L_1_ should be 50 mm. As shown in **Figures 10b**, with the increase of the length L_4_ of the rocker AB, the vertical distance between the lowest point of the relative movement track of the seedling clamping point of the transplanting device and the ridge surface gradually increases, and the transplanting depth increases accordingly. From the analysis of absolute movement trajectory, it is found that the angle between trajectory and horizontal direction decreases slightly with the increase of L_4_, so that, the transplanting inclination angle decreases slightly. Considering the influence of transplanting depth and angle, the value of L_4_ was determined to be 100 mm. According to the relative movement track shown in **Figures 10c**, with the increase of β angle, the relative movement track of seedling clamping point of transplanting device presents clockwise rotation trend, and the vertical distance between the lowest point of track and ridge surface gradually decreases, so that, the transplanting depth gradually decreases. Yet, the distance between the lowest point and the highest point remains unchanged, indicating that the movement amplitude of mechanism does not change due to the increase of β but only the overall displacement changes. From the analysis of absolute motion trajectory curve, it can be seen that the angle between trajectory and horizontal direction increases slightly with the increase of β, so that, the inclination angle increases. In order to ensure the transplanting verticality, β was determined to be 160°. According to the analysis of relative movement track shown in **Figures 10d**, with θ_5_ gradually increasing, the vertical distance between the lowest point of relative movement track of seedling clamping point of transplanting device and ridge surface decreases slightly, and the transplanting depth decreases slightly; From the absolute movement trajectory, it can be seen that the increase of θ_5_ has a little effect on the tilt angle during transplanting, so the value of θ_5_ is determined to be 85°.

### 3.2 Solution of optimum parameter combination of transplanting mechanism

**Figure 11:**
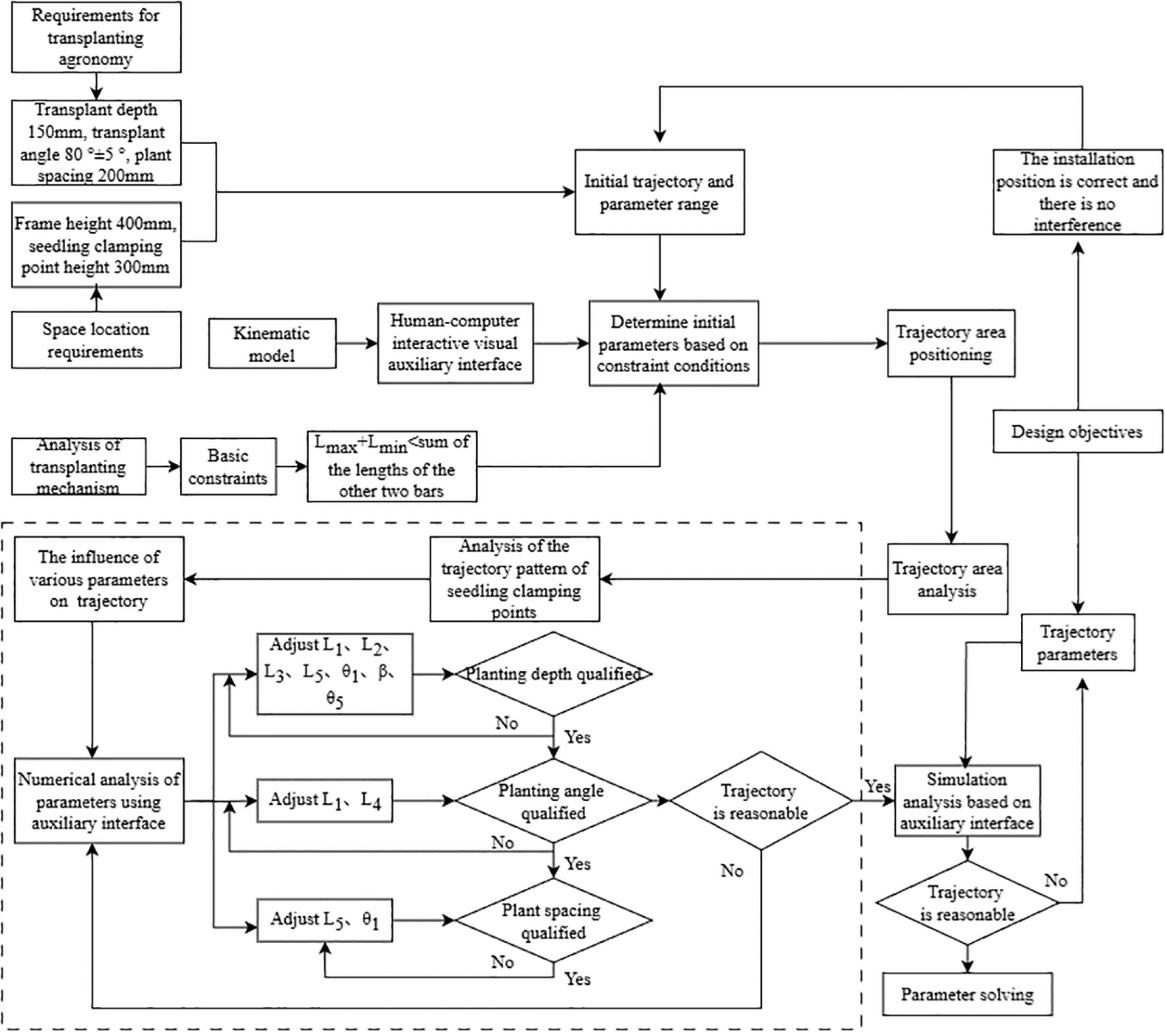
Conditional constraints and solution process.

Through MATLAB human-computer interaction visual interface, the optimal value of each parameter is obtained. The basis of solution is: the maximum transplanting depth is close to 150 mm; the angle α between the seedling clamping point and the horizontal plane at the lowest point of the trajectory is not less than 75°, and the closer is to 90°, the better is; the transplanting spacing is closest to 200 mm as much as possible. The optimal parameter combination satisfying the spoon shaped clamping mechanism chrysanthemum transplanting device is obtained as L_1_ = 50 mm, L_2_ = 110 mm, L_3_ = 130 mm, L_4_ = 100 mm, L_5_ = 150 mm, θ_1_ = 20°, β=160°, θ_5_ = 85°. The results also take into account the agronomic requirements, i.e. transplanting depth, seedling position and transplanting spacing. The solution process is shown in **Figure 11**, where the dashed box represents the parameter loop comparison for optimal parameter combination.

**Figure 12:**
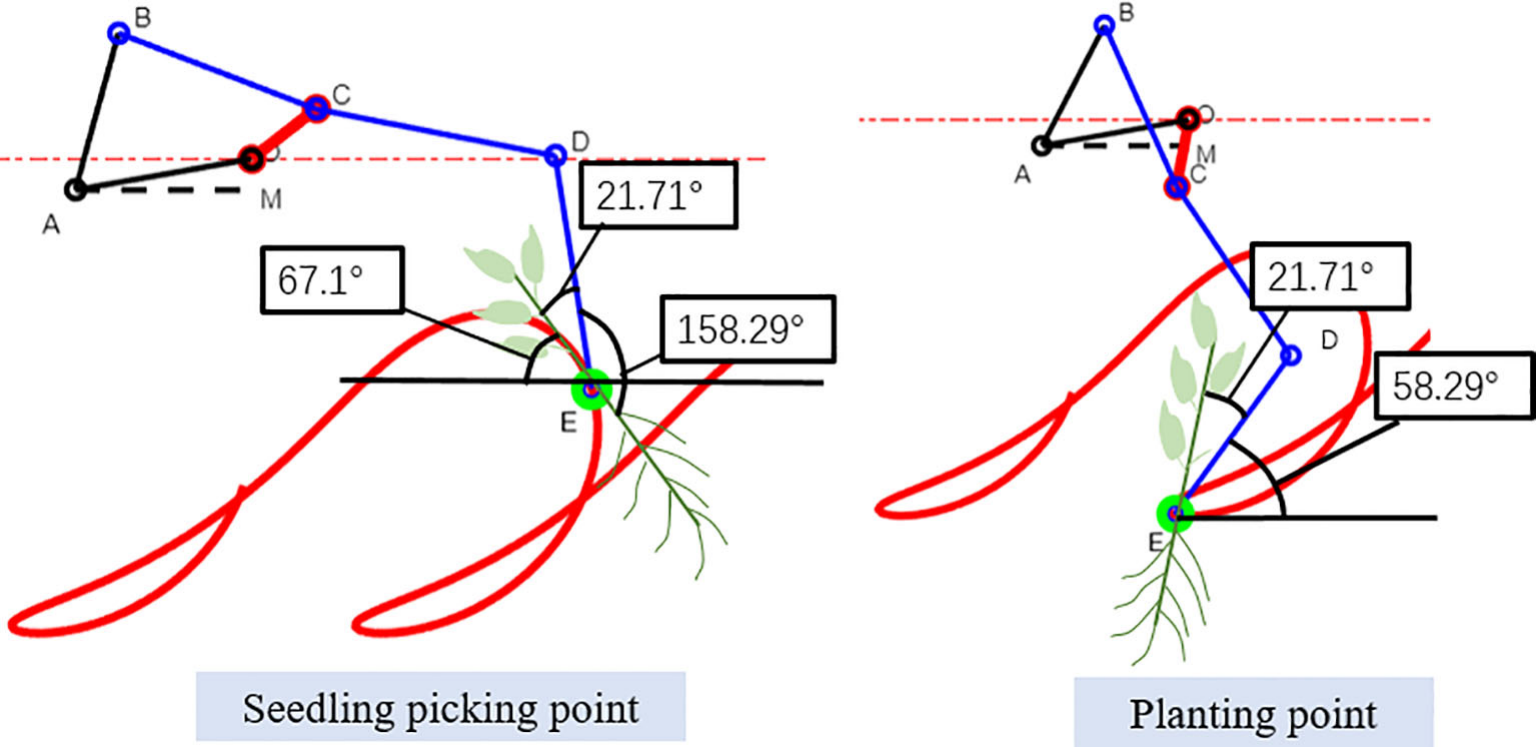
Spatial state of clamping mechanism and transplanted seedlings.

From the trajectory corresponding to the optimal parameter combination, the spatial relative state of the clamping mechanism and the transplanted seedlings can be extracted when the E point moves to the seedling picking point and the seedling releasing point (**Figure 12**). The position and angle data of the key points in this state can provide a basis for the design of the core components of the transplanting device.

Based on the analysis of visual interface, the relationship between transplanting device and chrysanthemum seedlings was obtained when transplanting device ran to seedling picking point and seedling releasing point. At the time of seedling picking, the angle between chrysanthemum seedling and DE axis of clamping mechanism is 21.71°, the angle between the center line of spoon shaped device enveloping roots and DE axis is 158.29°, while the actual angle is 158°. At this time, seedlings and clamping rubber clips fully fit together, the angle between rubber clamping surface and horizontal plane is 67.1°. When the transplanting device moves to the seedling releasing point, the angle between the clamping mechanism DE and the horizontal plane is 58.29°. In order to meet the requirement of 80° ± 5° between chrysanthemum seedling and horizontal plane, the angle between chrysanthemum seedling stem and DE axis should be kept at 21.71°.

## 4 Experiments and results

### 4.1 Experimental verification of transplanting trajectory

**Figure 13:**
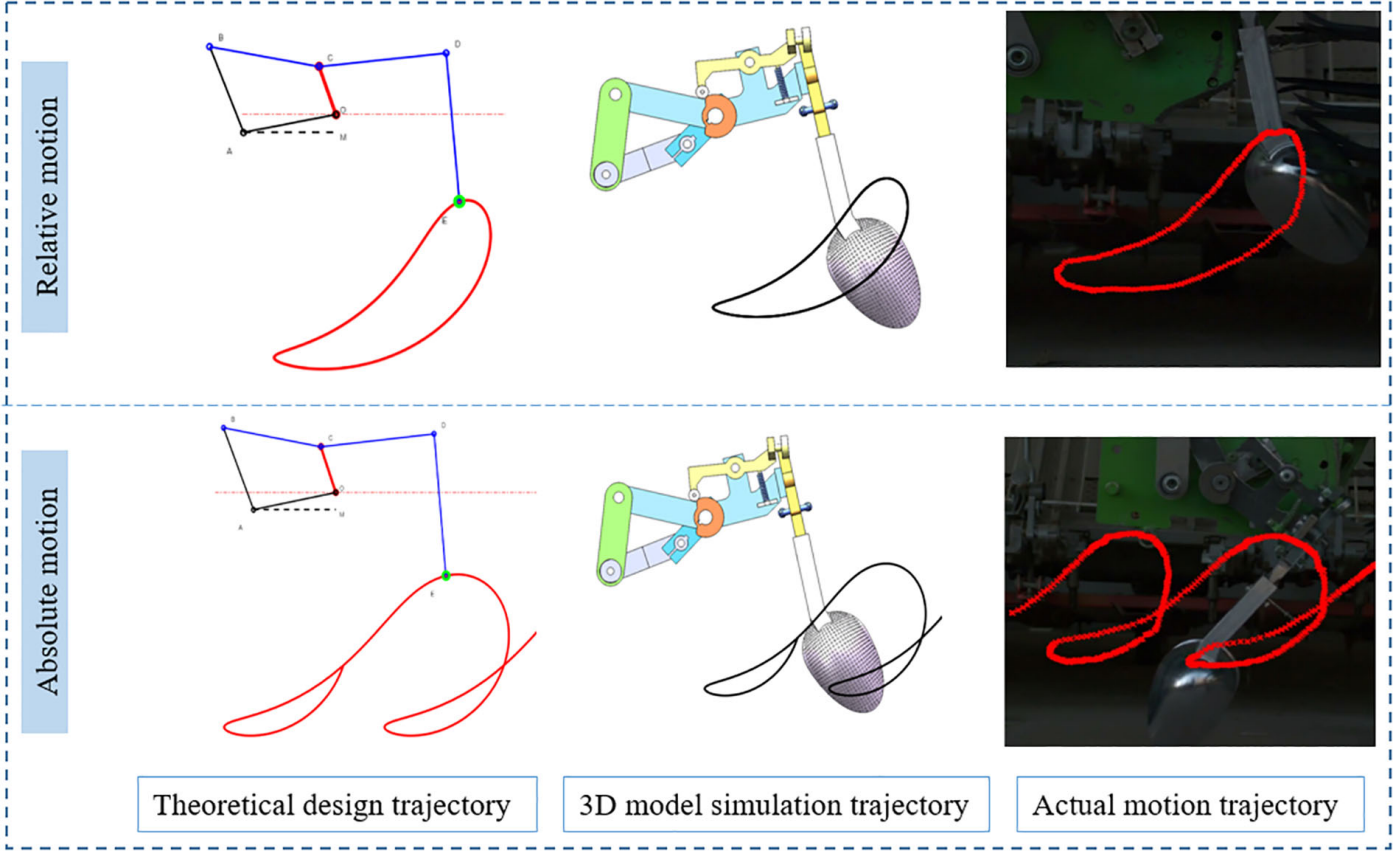
Comparison of motion trajectories.

This study used the Chronos 1.4 high-speed camera to capture and record the relative and absolute motion trajectories of the transplanting device. The i-SPEED Control analysis software was used to process the acquired high-speed video, the motion trajectory of the seedling clamping point E was extracted, and the marked points are connected and marked by red line segments, so as to extract the transplanting track formed by the marked points of the transplanting device. As shown in **Figure 13**, when the transplanting device performs relative motion and absolute motion, its theoretical design trajectory, three-dimensional model simulation trajectory and actual motion trajectory based on high-speed camera are in good agreement. The results show that the designed planting trajectory can be accurately reproduced in the actual movement, which verifies its feasibility and reliability.

### 4.2 Field experiment of transplanting device

**Figure 14:**
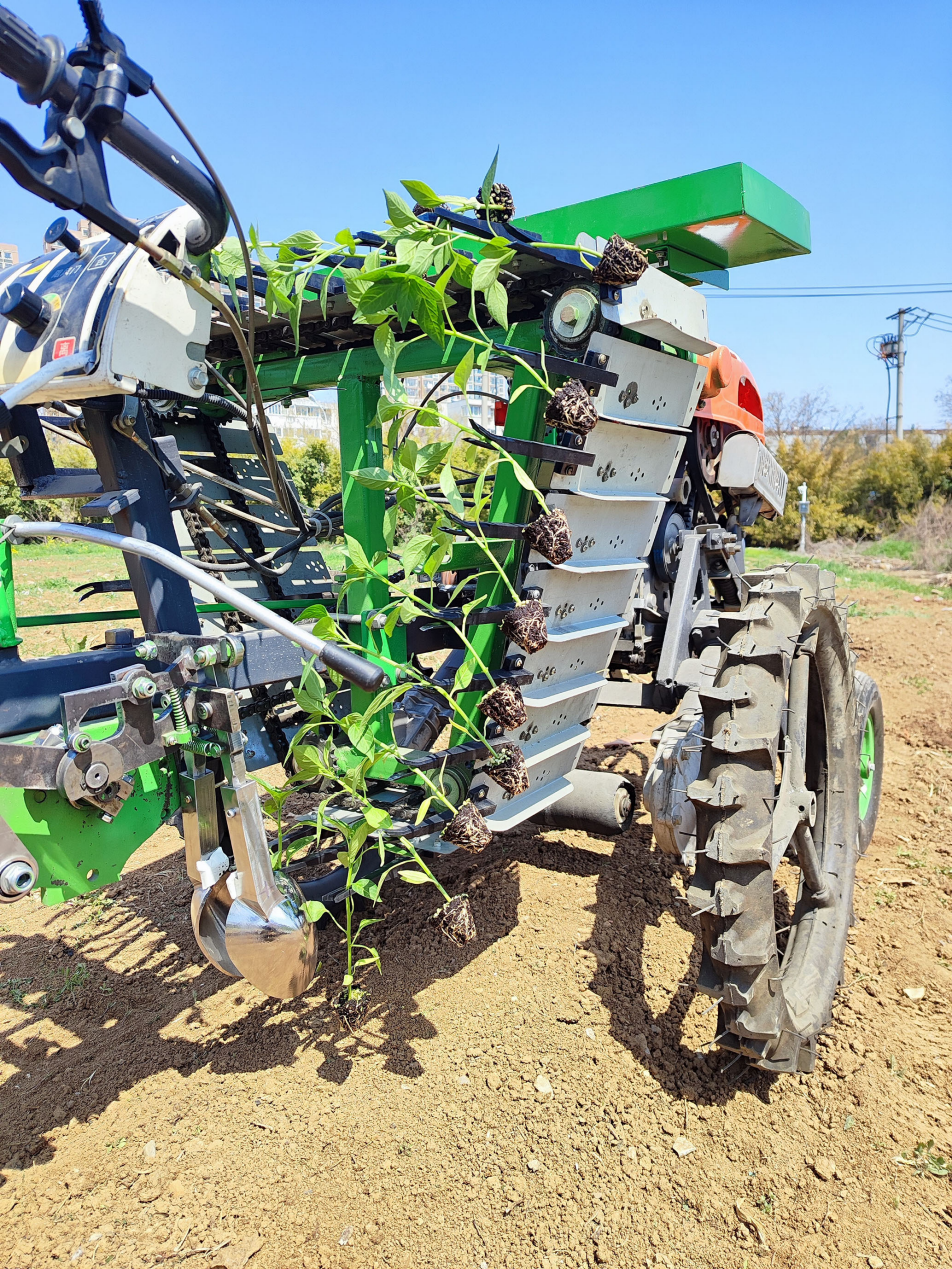
Field experiment of transplanting machine.

In order to verify the operation performance of spoon shaped clamping chrysanthemum seedling transplanting device, a field experiment was carried out at Anhui Agricultural University on March 22,2025. Before the experiment, artificial ridging was used, and the ridge size was as follows: height of 200 mm, bottom width of 400 mm, surface width of 300 mm. Because the normal transplanting period of chrysanthemum seedlings was 4~5 months, and the transplanted seedlings did not reach the suitable transplanting standard, pepper seedlings with similar morphology and size were selected as alternative experimental materials. The selected transplanted seedlings have an average height of 260 ± 21 mm and an average diameter of 5 ± 0.4 mm. In order to meet the agronomic requirement of transplanting spacing of 200 mm, the forward speed of the machine was set up at 0.19 m·s^-1^ and the crank rotation speed was 67 r·min^-1^. The field transplanting test is shown in **Figure 14**.

According to the Chinese standard for transplanting machinery operation JB/T T10291-2013, which is a mechanical industry standard issued by the Ministry of Industry and Information Technology of the People’s Republic of China, entitled “Quality of Transplant Machinery Operation”. The experiment adopted the scheme of transplanting 40 plants in each group and repeating 3 times. The measurement indexes include transplanting depth, angle and spacing, and the qualified rate of transplanting depth, the qualified rate of transplanting angle and the coefficient of variation (CV) of transplanting spacing are calculated accordingly as the main basis for evaluating transplanting performance (Wen et al., 2021; Pan et al., 2022; Jin et al., 2022). The method for measuring the transplanting depth is to gently dig up the soil on the side of the seedlings to avoid disturbing the original spatial position of the seedlings, and measure the vertical distance from the lowest point of the transplanted seedlings to the ridge plane. The measurement of transplanting angle needs to use angle ruler equipped with a digital display (range 0~360°) to measure the angle between the main stem of transplanted seedlings and the ridge plane. The horizontal distance between the stem insertion points of two adjacent transplanted seedlings was measured with a tape (range: 1~3m). After transplanting, transplanting angle was ≥75°, which is regarded as qualified. The ideal is 90°. The calculation method of qualified rate of transplanting angle is Equation 7:

(7) $$S_{A}=W_{A}/W_{Z}\times 100\%$$

Where *W*_A_ is the number of qualified plants in the transplanted seedlings, *W*_Z_ is the total number of transplanted plants.

Too deep or too shallow transplanting is not conducive to subsequent growth, so that the acceptable range of transplanting depth is 150 ± 20 mm. Equation 8 is used to calculate the qualified rate of transplanting depth:

(8) $$S_{D}=W_{D}/W_{Z}\times 100\%$$

Where, *W*_D_ is the number of plants with qualified transplanting depth.

The coefficient of variation (CV) of transplanting spacing can reflect the uniformity of transplanting spacing distribution. Firstly, it was needed to calculate the average value of transplanting spacing (Equation 9) and the standard deviation of transplanting spacing (Equation 10), and, then, the coefficient of variation of transplanting spacing (Equation 11):

(9) $$\bar{X}=\frac{\sum_{i=1}^{n}X_{i}}{n}$$

(10) $$S_{X}=\sqrt{\frac{1}{n-1}\sum {\left(X_{i}-\bar{X}\right)}^{2}}$$

(11) $$\text{C}V_{X}=S_{X}/\bar{X}\times 100\%$$

where *X*_i_ is the i-th transplanting spacing measured value, and *n* is the sample number.

### 4.3 Experiment data and analysis

The results of transplanting experiments are shown in **Table 4**. When the crank rotation speed is 67 r·min^-1^ and machine forward speed is 0.19 m·s^-1^, the average qualified rate of transplanting depth is 90.0%, the average qualified rate of transplanting angle is 84.2%, and the coefficient of variation (CV) of transplanting spacing is 5.8%. The experiment results showed that the transplanting machine met the agronomic requirements of chrysanthemum transplanting, and all indexes basically reached the standard of transplanting machine on dry soil.

**Table 4:** Results of transplanting experiment.

Number	Qualified rate of transplanting angle/%	Qualified rate of transplanting depth/%	Coefficient of variation (CV) of transplanting spacing/%
1	90.0	90.0	5.1
2	82.5	92.5	5.8
3	80.0	87.5	6.6
Average/%	84.2	90.0	5.8

**Figure 15:**
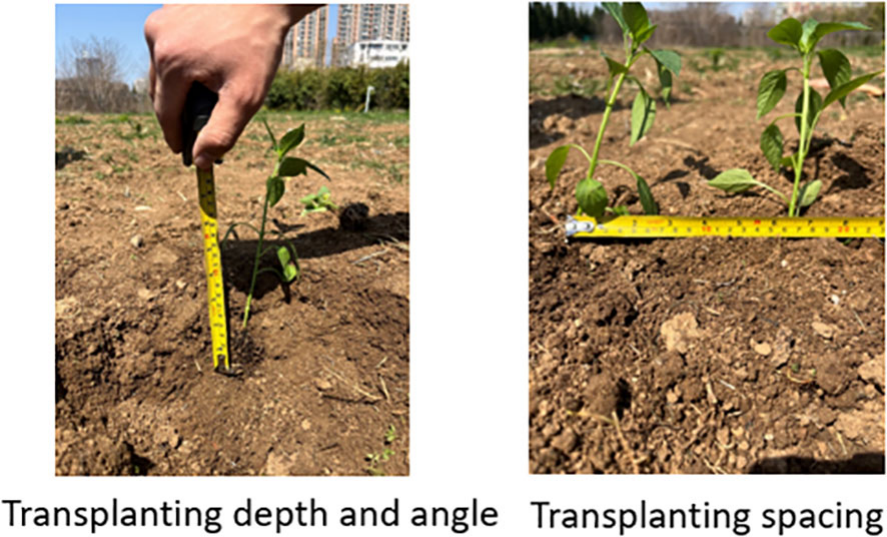
Measurement of transplanting performance.

From the transplanting performance shown in **Figure 15**, it can be seen that the seedling is enough erect, the transplanting spacing is uniform, and the overall transplanting quality is high.

### 4.4 Discussion

Compared to existing transplanting mechanisms, the proposed spoon shaped clamping mechanism demonstrates several significant distinctions and improvements in performance. Although limited by experimental conditions, this study did not provide more step-by-step photos. However, the trajectory comparison and data analysis sufficiently demonstrate the motion stability and trajectory controllability of the spoon-shaped clamping mechanism during the transplanting process. Firstly, addressing the issues of obvious missing seedlings associated with chain clamp type, this study achieves coordinated protection and placement of the seedling’s root stem system through the spoon component cradling the root and the flexible clamp securing the stem. This is reflected in the good spacing uniformity in the tests. Secondly, targeting the problem of root and stem damage caused by gripper type mechanisms, this study designed a combination of enveloping spoon structure and rubber clamp to distribute clamping force, significantly reducing the risk of mechanical damage to the stem. Furthermore, compared to the widely used duckbill type, this study through optimized linkage parameters and trajectory, achieved a high transplantation angle qualification rate of 84.2%. However, it must be acknowledged that clamp loosening due to machine vibration remains a primary limitation affecting the angle qualification rate and spacing consistency, a challenge related to the dynamic balance of multi-link mechanisms at operational speeds. Due to the vibration produced by the whole machine, that makes the seedlings slightly lose in the rubber clip. When the spoon shaped clamping mechanism is opened, the seedlings fail to reach the set up position, resulting in deviation of clamping angle, which affects the accuracy of transplanting angle. At the same time, the vibration of the machine also reduces the stability of the clamping device, which affects the consistency of transplanting spacing. Future structural optimizations for this study may include: first, dynamic balancing design and correction of key rotating components (e.g., cranks, cams); second, the use of rubber or polyurethane damping pads at the connections between the frame and the transplanting device to absorb high-frequency vibrations; additionally, damping structures could be added between the swing push rod spring and the opening and closing spring to reduce clamp loosening. These measures are expected to effectively improve the qualified rate of transplanting angle and the consistency of planting spacing without significantly increasing costs.

The transplanting mechanism addresses a research gap in specialized equipment for the mechanical transplanting of chrysanthemums. It strikes a balance between cost, complexity and performance, offering a viable solution for small to medium-scale operations. Its core advantage lies in good adaptability to specific crops like chrysanthemums, which with multiple fibrous roots. It protects the root akin to spoon shaped mechanisms, while overcoming the seedling tilting during planting phases through active stem stabilization. Future work should focus on suppressing vibration through structural optimization to further enhance operational stability and qualification rates.

## 5 Conclusions

1. Based on the physical characteristics and agronomic requirements of chrysanthemum seedlings, a spoon shaped clamping transplanting device was designed, and its working principle and kinematics model were described. The mechanism can realize the seedling picking, clamping and planting, and effectively solves the problem of adaptability of traditional transplanting machines to chrysanthemum seedlings.

2. Based on MATLAB platform, the human-computer interactive visual analysis software of transplanting device was developed, and 8 key parameters were optimized and analyzed. The results showed that OC length L_1_, AB length L_4_, BC and CD angle β, CD and DE angle θ_5_ had dominant influence on transplanting spacing, depth and angle. The optimal parameter combination is obtained by trajectory simulation: L_1_ = 50 mm,L_2_ = 110 mm,L_3_ = 130 mm,L_4_ = 100 mm,L_5_ = 150 mm,θ_1_ = 20°,β=160°,θ_5_ = 85°. The trajectory of seedling clamping points obtained by high-speed camera is consistent with the simulated trajectory and theoretical design trajectory, which verifies the rationality of transplanting trajectory design.

3. The prototype field experiment was carried out by means of the optimized transplanting device. Under the operating conditions of crank rotation speed of 67 r·min^-1^ and machine forward speed of 0.19 m·s^-1^, the average qualified rate of transplanting depth was 90.0%, the average qualified rate of transplanting angle was 84.2%, and the coefficient of variation (CV) of transplanting spacing was 5.8%. Therefore, the transplanting performance of this mechanism meets the agronomic requirements of chrysanthemum seedling transplanting.

## Data Availability

The original contributions presented in the study are included in the article/supplementary material. Further inquiries can be directed to the corresponding author.
